# Hyperthyroidism and human chorionic gonadotrophin production in gestational trophoblastic disease

**DOI:** 10.1038/bjc.2011.139

**Published:** 2011-04-26

**Authors:** L Walkington, J Webster, B W Hancock, J Everard, R E Coleman

**Affiliations:** 1 Academic Unit of Clinical Oncology, Weston Park Hospital, Sheffield S10 2SJ, UK; 2 Department of Endocrinology, Royal Hallamshire Hospital, Sheffield S10 2JF, UK

**Keywords:** hyperthyroidism, GTD, HCG

## Abstract

**Background:**

Gestational trophoblastic disease (GTD) is a rare complication of pregnancy, ranging from molar pregnancy to choriocarcinoma. Patients with persistent disease require treatment with chemotherapy. For the vast majority, prognosis is excellent. Occasionally, GTD is complicated by hyperthyroidism, which may require treatment. This is thought to occur due to molecular mimicry between human chorionic gonadotrophin (HCG) and thyroid-stimulating hormone (TSH), and hence cross-reactivity with the TSH receptor. Hyperthyroidism usually resolves as the GTD is successfully treated and correspondingly HCG levels normalise.

**Methods:**

This paper reviews cases of GTD treated over a 5-year period at one of the three UK centres and identifies the prevalence of hyperthyroidism in this population. Four cases with clinical hyperthyroidism are discussed.

**Results:**

On review of the 196 patients with gestational trophoblastic neoplasia treated with chemotherapy in Sheffield since 2005, 14 (7%) had biochemical hyperthyroidism. Of these, four had evidence of clinical hyperthyroidism.

**Conclusion:**

Concomitant biochemical thyroid disease in patients with GTD is relatively common, and measurement of thyroid function in patients with persistent GTD is, therefore, important. The development of hyperthyroidism is largely influenced by the level of HCG and disease burden, and usually settles with treatment of the persistent GTD. However, rarely the thyroid stimulation can have potentially life-threatening consequences.

Gestational trophoblastic disease (GTD) is an uncommon complication of pregnancy with an incidence in the United Kingdom of approximately 1.5/1000 births (Bagshawe *et al*, 1986). Gestational trophoblastic disease is essentially a family of diseases that includes complete and partial molar pregnancy, choriocarcinoma and placental site trophoblastic tumour. Choriocarcinoma is a malignant form of GTD, occurring in approximately 1 : 70000 pregnancies per year, and may occur after a normal pregnancy (post-partum choriocarcinoma) (Ober *et al*, 1971).

In the United Kingdom, all women with molar pregnancies are registered at one of the three dedicated GTD centres (London, Sheffield, Dundee) for ongoing monitoring and treatment if necessary. The centre in Sheffield receives 500–600 registrations each year. Approximately 6–8% of women subsequently require treatment, usually chemotherapy, for persistent disease (gestational trophoblastic neoplasia, GTN). The prognosis is excellent with a cure rate in excess of 95% (Fisher and Hancock, 1997).

There is well-documented evidence of the association between high serum human chorionic gonadotrophin (HCG) levels and suppressed thyroid-stimulating hormone (TSH) in normal pregnancy (Braunstein and Hershman, 1976; Glinoer *et al*, 1990), often in the context of hyperemesis gravidarum (Goodwin *et al*, 1992). Less often, this association is seen in gestational neoplasia and other germ cell malignancies in which HCG is secreted. Often in these cases, this is an incidental finding, the free T3 (FT3) and free T4 (FT4) are within normal limits, and the patient is asymptomatic. Patients rarely require any treatment, and the thyroid function tests (TFTs) normalise as the serum HCG falls. The effect on the thyroid is thought to occur due to molecular mimicry between HCG subunits and TSH (Fisher and Hancock, 1997). The potency of HCG for TSH receptors is some 4000 times less than TSH and hence, extremely high levels of HCG are usually required for an effect on thyroid function to be seen.

There have been a small number of case reports of GTD presenting in association with symptomatic hyperthyroidism (Odell *et al*, 1963; Morley *et al*, 1976; Anderson *et al*, 1979; Norman *et al*, 1981; Soutter *et al*, 1981).

Here, we review TFT results in patients treated at our centre over the past 5 years, and report on four patients with clinical hyperthyroidism.

## Materials and methods

All chemotherapy prescribed at Weston Park Hospital is done through Chemocare, an electronic prescribing system. This system was used to create a database of all patients commencing chemotherapy for GTN between January 2005 and January 2010. The diagnoses of the patients treated were confirmed by cross-referencing with their medical notes and their biochemical markers (HCG, TSH, FT3 and FT4) were obtained from our computerised results recording system.

## Results

**Figure 1:**
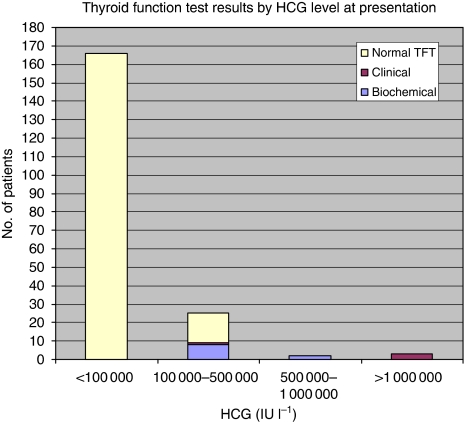
Frequency of hyperthyroidism by HCG level in patients treated in Sheffield January 2005 to January 2010. *Note*: Clinical hyperthyroidism defined as TSH <0.03 mIU l^–1^ and signs/symptoms consistent with hyperthyroidism (tachycardia, tremor and breathlessness) requiring treatment with anti-thyroid drugs (one case with HCG 100 000–500 000 IU l^–1^ at presentation and three cases with HCG>1 mIU l^–1^). Biochemical hyperthyroidism defined as TSH <0.03 mIU l^–1^ without any clinical signs/symptoms of hyperthyroidism and not requiring active treatment.

On review of the 196 patients with GTN treated with chemotherapy in Sheffield since 2005, 14 (7%) had biochemical hyperthyroidism. Of these, four had evidence of clinical hyperthyroidism. All patients had HCG levels of >100 000 IU l^–1^. Most patients had HCG levels between 100 000 and 500 000 IU l^–1^ at presentation (Figure 1).

Case 1 was a usually well, 22-year-old female who, following an uncomplicated pregnancy, presented 24 days after the normal vaginal term delivery of her third child. The delivery was complicated by a retained placenta, which required manual evacuation. She subsequently presented twice to her local hospital with heavy vaginal bleeding, breathlessness and tachycardia. She underwent a surgical evacuation of her uterus, although no specimens were sent for histology. Her serum HCG was found to be 1.077 × 10^6^ IU l^–1^ (normal range <2 IU l^–1^). Chest X-ray showed multiple cannon-ball lesions and a presumed diagnosis of post-partum choriocarcinoma was made.

On transfer to our centre, she was unwell with haemodynamic instability, breathlessness, fever and abdominal pain with distension. Further staging investigations confirmed multiple lung metastases, and pelvic ultrasound showed an 18 × 8 × 8 cm^3^ intrauterine mass. Magnetic resonance imaging (MRI) of the brain was normal. She was calculated to have a World Health Organisation (WHO) risk score of 12 (Kohom, 2001) (Table 1) and commenced on first-line high-risk chemotherapy with intravenous methotrexate, alternating with intravenous dactinomycin and etoposide (IV MAE).

As per our local policy, her thyroid function was measured at time of presentation. She was found to have hyperthyroidism with TSH <0.03 mIU l^–1^ (normal range 0.35–4.5 mIU l^–1^), FT3 6.5 pmol l^–1^ (normal range 3.5–6.5 pmol l^–1^) and FT4 36.3 pmol l^–1^ (normal range 10.0–19.8 pmol l^–1^). Thyroid auto-antibodies were negative. She was commenced on carbimazole 40 mg daily (OD) and propranolol 40 mg three times daily prior to commencement of chemotherapy.

**Figure 2:**
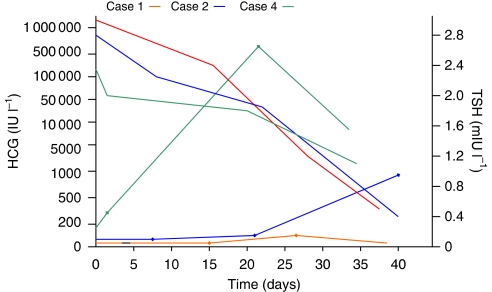
HCG and TSH trends over time.

She responded quickly to chemotherapy with rapid falls in serum HCG (Table 2; Figure 2). She was clinically and biochemically euthyroid by commencement of her third cycle of chemotherapy. Her carbimazole and propranalol were steadily reduced and subsequently stopped. Her HCG level has now normalised, she has completed chemotherapy and is clinically well, with clinical and biochemical euthyroidism off all treatment.

Case 2 was a 21-year-old woman who presented to her local hospital with abdominal pain and haemodynamic instability, while taking the oral contraceptive pill. A pregnancy test was positive, but ultrasound of the pelvis revealed an empty uterus. She underwent a right salpingetcomy for a presumed ruptured ectopic pregnancy. She was commenced on depo-contraceptive immediately following her surgery. Two months following her ectopic pregnancy, she re-presented with abdominal pain and a raised serum HCG of 140 000 IU l^–1^. A pelvic ultrasound showed an empty uterus and a complex mass arising in the right side of the pelvis. A pelvic MRI scan confirmed that this was arising adjacent to or within the right ovary.

She was transferred to Sheffield and on arrival was breathless at rest with a resting tachycardia. A presumptive diagnosis of choriocarcinoma was made and this was later confirmed on review of the histology from her ruptured ectopic pregnancy, which initially had not been reported as such. Further staging investigations including chest imaging and MRI of the brain showed no evidence of metastatic disease. Serum HCG following transfer was 1.176 × 10^6^ IU l^–1^. She had a WHO risk score of 7 and was commenced on IV MAE.

Thyroid function tests showed her to be hyperthyroid, with TSH <0.03 mIU l^–1^, FT4 73 pmol l^–1^ and FT3 21.6 pmol l^–1^, in keeping with her symptoms of breathlessness, agitation and resting tachycardia. She was commenced on carbimazole and propranolol.

After her first cycle of treatment, she developed severe abdominal pain and swelling. This was associated with a dramatic fall in serum albumin. Pelvic MRI revealed ascites and a complex right ovarian mass. A diagnosis of ovarian hyperstimulation syndrome was made. She was managed conservatively and her symptoms, signs and serum albumin levels normalised over the following 10 days with further chemotherapy.

Her TFTs normalised in parallel with the fall in serum HCG (Table 2; Figure 2). She was slowly weaned off her anti-thyroid treatment and noted to be biochemically euthyroid at commencement of her third cycle of chemotherapy. She has now completed chemotherapy, is well with normal serum HCG and remains clinically and biochemically euthyroid.

Case 3 was a 45-year-old woman who presented 6 weeks after evacuation of a molar pregnancy detected at her obstetric dating scan. Histology showed a complete hydatiform mole. Serum HCG on presentation to our centre was 2.445 × 10^6^ IU l^–1^. Her staging investigations showed multiple lung metastases. Her WHO risk score was 8, and she commenced on IV MAE.

Fourteen days after her first cycle of treatment, she presented acutely breathless. Her serum HCG had fallen by more than half (942 331 IU l^–1^), but she was noted to have a suppressed TSH (<0.03 mIU l^–1^) with normal FT4 (19 pmol l^–1^) and FT3 (6.1 pmol l^–1^). Her albumin had fallen dramatically (23 g l^–1^). She was noted to have signs and symptoms of acute heart failure and an echocardiogram showed mild-to-moderate left ventricular impairment. She was treated symptomatically with diuretics and commenced on *β*-blockers. She did not require specific treatment for her abnormal TFTs, and these normalised with successful treatment of her GTD. Thyrotoxicosis may have been the cause of her heart failure, although the differential diagnosis includes idiopathic post-partum heart failure. Whatever the cause, her cardiac function subsequently normalised both clinically and as evidenced on serial echocardiograms.

After several cycles of chemotherapy, the fall in her HCG levels plateaued at around 100 IU l^–1^. She subsequently had a hysterectomy, following which her serum HCG fell to normal. She remains well with no evidence of active disease, is clinically and biochemically euthyroid and has normal cardiac function.

Case 4 was a 51-year-old woman who presented to her GP with a 6-month history of lethargy, flushes, abdominal distension and irregular vaginal bleeding. On examination, she was noted to have a palpable goitre and a central abdominal mass. Thyroid function tests showed her to be mildly hyperthyroid with TSH <0.03 mIU l^–1^, FT3 6.7 pmol l^–1^ and FT4 16.7 pmol l^–1^.

She was commenced on carbimazole 20 mg OD. A computerised tomography (CT) scan of the abdomen showed a 14 × 18 cm^2^ intrauterine mass. While awaiting specialist referral, she presented to her local accident and emergency department where she spontaneously delivered a large mass of jelly-like tissue and blood from the vagina. Histology showed a complete mole. Five days later, she was transferred to our centre. Serum HCG was 115 942 IU l^–1^. Computerised tomography chest showed multiple small lung metastases. Magnetic resonance imaging of the brain was normal. Her WHO risk score was 5, putting her into a low-risk category for which she was commenced on intra-muscular methotrexate.

At commencement of her chemotherapy, and after 4 weeks of carbimazole, her TSH was 0.003 mIU l^–1^, FT4 12.9 pmol l^–1^ and FT3 3.6 pmol l^–1^. Her TFTs parallelled the fall in her HCG, and were normal by the start of her second cycle of chemotherapy (Table 1; Figure 2). Her carbimazole was slowly reduced and subsequently stopped. Due to an incomplete response to methotrexate, she required a change of chemotherapy to single agent intravenous dactinomycin. Her HCG level is now normal, she is well and clinically and biochemically euthyroid.

## Discussion

Subclinical hyperthyroidism is well documented in pregnancy, but clinical manifestations are uncommon. At the time of peak HCG levels in normal pregnancy, serum TSH falls and bears a mirror image to the HCG peak. This fall in TSH suggests that it is HCG that causes increased secretion of T3 and T4 (Hershman, 2004). When there are clinical features of thyrotoxicosis, as in some cases of hyperemesis gravidum, anti-thyroid treatment may be given, but is rarely required beyond 22 weeks gestation.

Human chorionic gonadotrophin is a glycoprotein composed of *α* and *β* subunits. The *α* subunit is almost identical to that found in TSH, luteinising hormone (LH) and follicle-stimulating hormone (Hershman, 2004). The subunit consists of a 92-amino-acid chain containing two nitrogen-linked oligosaccharide side chains. *In vitro* testing has shown low-affinity cross-reactivity between these hormones (Vaitukaitis, 1977; Hershman, 2004). The HCG subunits all target one or more of the G-protein-coupled seven transmembrane receptors, and have a high degree of homology in their transmembrane domains (Vassart and Dumont, 1992). The LH/HCG receptors share 45% homology with the TSH receptor (Nagayama and Rapoport, 1992; Kohn *et al*, 1995).

Hyperthyroidism (defined as a suppressed TSH with raised FT3 or FT4) is more common in trophoblastic disease than normal pregnancy. It is thought that the HCG produced in woman with GTD has enhanced thyrotrophic activity compared with HCG in normal pregnancy. This is supported by the observation that cAMP production is increased more by HCG associated with GTD when transfected with human TSH-R on Chinese hamster ovary cells (Kato *et al*, 2004). When compared with human TSH, studies using cAMP have identified the potency of purified HCG to be 0.72 *μ*U per UHCG (Hershman *et al*, 1998).

This low potency of HCG for TSH receptors is reflected clinically when hyperthyroidism is seen in GTN because clinical manifestation occurs only in the context of very high levels of HCG. Previous case studies have indicated that serum levels of HCG of >100 000 mIU l^–1^ are usually needed to produce clinical evidence of thyrotoxicosis (Odell *et al*, 1963; Morley *et al*, 1976; Anderson *et al*, 1979; Norman *et al*, 1981; Soutter *et al*, 1981).

In our patient cohort, three cases had serum HCG in excess of 1 million at presentation. In case 4, it is likely that her serum HCG had begun to fall rapidly following spontaneous passage of the molar tissue, and had the serum HCG been checked when her hyperthyroidism was diagnosed, the HCG may have been much higher.

The level of thyroid stimulation is directly proportional to HCG concentration. Therefore, the severity of clinical hyperthyroidism also reflects the HCG level. Human chorionic gonadotrophin thyrotropic activity may also be influenced by metabolism of HCG molecule; deglycosylation and/or desialylation of HCG may enhance its thyrotropic properties (Norman *et al*, 1981).

In the United Kingdom, the co-ordinated and centralised management of GTN means that patients usually present early and as such very high levels of HCG are unusual, and thus hyperthyroidism, especially in a clinically overt form, is rare. This may not be the case in healthcare systems, which do not enable early diagnosis.

The largest case series of women with GTD and thyroid disease comes from Africa. In 27 patients with GTD, more than half (15 out of 27) were biochemically hyperthyroid (8 choriocarcinoma and 7 hydatidiform mole) at presentation. Of these, 60% were clinically thyrotoxic and one developed acute pulmonary oedema with associated heart failure. The patients were all euthyroid once HCG fell to <30 000. It was noted that T4 was raised invariably, but the T3:T4 ratio was low (0.015+/−0.005) (Norman *et al*, 1981).

This seems to be a higher proportion of the patients than observed in our practice. This could reflect the racial difference between the cohorts, or differences in health care provision leading to the women in Africa presenting later in their disease and with higher HCG levels.

When hyperthyroidism occurs in the context of choriocarcinoma, case reports have demonstrated that the biomarkers of choriocarcinoma and thyroid function parallel the regression and subsequent relapse of the tumour (Hsieh *et al*, 2008). This was also observed in our patients who all responded rapidly to chemotherapy.

Most patients with symptomatic hyperthyroidism respond rapidly to treatment. However, there have been a few case reports of patients with thyroid storm, such as a 17-year-old woman presenting hyperthyroid with haemodynamic compromise and a previously undiagnosed molar pregnancy (Moskovitz and Bond, 2009). She required intensive care support, but was treated successfully with *β*-blockers and propylthiouracil for her thyroid disease and underwent a surgical evacuation of her molar pregnancy, which then required no further treatment. Paradoxically, severe thyrotoxicosis has also been reported after commencing treatment of GTD. A 53-year-old woman developed profound cardiovascular instability 1 day after surgical evacuation of a complete hydatiform mole. She was not known to have pre-existing thyroid disease, but investigations confirmed thyrotoxicicosis (Struthmann *et al*, 2009).

Finally, although there is significant evidence of the cross-over effects of HCG and TSH, and demonstration of thyrotropic activity of HCG, it should be noted that concomitant production of thyrotropin by the tumour cannot be excluded (Cooper *et al*, 1978). There are no published data regarding this as yet.

## Conclusions

Concomitant biochemical thyroid disease in patients with GTD is relatively common. Measurement of thyroid function in patients with persistent GTD is, therefore, important. The development of hyperthyroidism is largely influenced by the level of HCG and disease burden. Extremely high levels of HCG are typically required for the development of clinical hyperthyroidism as the relative potency of HCG for the TSH receptor is low. As a result, only a minority are clinically hyperthyroid and require treatment, although rarely the thyroid stimulation can have potentially life-threatening consequences. In those requiring treatment, carbimazole and *β*-blockers for symptom relief appear to be effective, and thyroid function can be expected to normalise rapidly with treatment of the underlying GTD and the consequent fall in HCG levels.

## Figures and Tables

**Table 1:** WHO prognostic scoring system for GTD

	**Score**			
**Prognostic factor**	**0**	**1**	**2**	**4**
Maternal age (years)	<40	⩾40		
AP	Mole	Abortion	Term	
Interval (end of AP to chemotherapy in months)	<4	4–6	7–13	>13
HCG IU l^–1^	<10^3^	10^3^–10^4^	10^4^–10^5^	>10^5^
Number of metastases	0	1–4	5–8	>8
Site of metastases	Lung	Spleen, kidney	GI tract	Brain, liver
Largest tumour mass		3–5 cm	>5 cm	
Prior chemotherapy			Single drug	⩾Two drugs

Abbreviations: AP=antecedent pregnancy; GI=gastrointestinal; GTD=gestational trophoblastic disease; HCG=human chorionic gonadotrophin; WHO=World Health Organisation.

**Table 2:** Summary of serial HCG and TFT measurements

	**Day**	**HCG (IU l^–1^)**	**TSH (mIU l^–1^)**	**FT4 (pmol l^–1^)**	**Carbimazole**
Case 1	0	1 077 249	<0.03	36.3	40 mg OD
	16	265 008	<0.03	20.7	20 mg OD
	27	2383	0.14	12.1	10 mg OD
	38	766	1.2	11.2	Stopped
					
Case 2	0	791 711	<0.03	73	40 mg OD
	7	156 420	<0.03	15.2	40 mg OD
	21	19 986	0.15	16.1	20 mg OD
	42	333	0.83	12.2	Stop
					
Case 4	0	115 942	0.03	12.9	20 mg OD
	2	69 604	0.05	12.2	10 mg OD
	19	47 966	2.5	6.7	10 mg OD
	33	4315	1.5	9.8	Stop

Abbreviations: FT4=free T4; HCG=human chorionic gonadotrophin; OD=once daily; TFT=thyroid function test; TSH=thyroid-stimulating hormone.
